# Systemic production of nitric oxide during severe asthma: immonumodulation by the helminth *echinococcus granulosus*

**DOI:** 10.1186/2045-7022-5-S2-P17

**Published:** 2015-03-23

**Authors:** Manel Amri, Merzak Gharnaout, Chafia Touil-Boukoffa

**Affiliations:** 1 University of Sciences and Technology Houari Boumediene (USTHB), Department of Cellular and Molecular Biology (BCM), Algiers, Algeria; 2 Rouiba Hospital, Department of pneumology, Algiers, Algeria

## Background

Several studies highlight the implication of nitric oxide (NO) in the airways inflammation during asthma. Moreover, asthma is thought to be associated with a systemic inflammation. However, little is known about the systemic production of NO in asmathic patients. In this sense, our aim was to evaluate the in vivo and ex vivo production of NO in Algerian patients with severe asthma. Moreover, the effect of helminthes infection on asthma is still inconclusive. In this sense, the second part of this study aims to investigate the effect of the helminth Echinococcus granulosus on this production. This parasite causes cyst formation particularly in lungs and liver.

## Method

The NO production was evaluated in sera and culture performed with mononuclear cells (PBMC) from asthmatic patients and healthy donors. We have also investigated the effect of laminated layer extract (LLs, outside layer of parasitic cyst) on NO production by the same cells.

## Results

We observed that in vivo and ex vivo NO levels assessed in patients with severe asthma are higher than those observed in healthy donors. Interestingly, we found that LLs reduced NO production ex vivo. This result was confirmed using L-NMMA, an inhibitor of nitric oxide synthases (NOS).

## Conclusion

Our data confirm the implication of NO in the pathogenesis of severe asthma. They also support the hypothesis that helminthes infection prevents and/or modulates inflammation during inflammatory diseases like asthma. The potentiel effect of laminated layer to reduce the inflammation in the airways and to reduce the risk of severe asthma remains to be investigated in mouse model of asthma.

